# Autophagy in the Human Placenta throughout Gestation

**DOI:** 10.1371/journal.pone.0083475

**Published:** 2013-12-13

**Authors:** Tai-Ho Hung, T’sang-T’ang Hsieh, Szu-Fu Chen, Meng-Jen Li, Yi-Lin Yeh

**Affiliations:** 1 Department of Obstetrics and Gynecology, Chang Gung Memorial Hospital, Taipei, Taiwan; 2 Department of Chinese Medicine, College of Medicine, Chang Gung University, Taoyuan, Taiwan; 3 Department of Physical Medicine and Rehabilitation, Cheng Hsin Rehabilitation Medical Center, Taipei, Taiwan; Medical Faculty, Otto-von-Guericke University Magdeburg, Medical Faculty, Germany

## Abstract

**Background:**

Autophagy has been reported to be essential for pre-implantation development and embryo survival. However, its role in placental development and regulation of autophagy during pregnancy remain unclear. The aims of this study were to (1) study autophagy by characterizing changes in levels of beclin-1, DRAM, and LC3B in human placenta throughout gestation; (2) determine whether autophagy is involved in regulation of trophoblast invasion in JEG-3 cells (a choriocarcinoma cell line); (3) examine the effects of reduced oxygen and glucose on the autophagic changes; and (4) investigate the effect of reoxygenation and supplementation of glucose after oxygen-glucose deprivation (OGD) on the autophagic changes in primary cytotrophoblasts obtained from normal term pregnancy.

**Methodology/Principal Findings:**

An analysis of 40 placental samples representing different gestational stages showed (1) no significant differences in beclin-1, DRAM, and LC3B-II levels in placentas between early and mid-gestation, and late gestation with vaginal delivery; (2) placentas from late gestation with cesarean section had lower levels of LC3B-II compared to early and mid-gestation, and late gestation with vaginal delivery; levels of DRAM were also lower compared to placentas from early and mid-gestation; and (3) using explant cultures, villous tissues from early and late gestation had similar rates of autophagic flux under physiological oxygen concentrations. Knockdown of *BECN1*, *DRAM*, and *LC3B* had no effects on viability and invasion activity of JEG-3 cells. On the other hand, OGD caused a significant increase in the levels of LC3B-II in primary cytotrophoblasts, while re-supplementation of oxygen and glucose reduced these changes. Furthermore, there were differential changes in levels of beclin-1, DRAM, and LC3B-II in response to changes in oxygen and glucose levels.

**Conclusions/Significance:**

Our results indicate that autophagy is involved in development of the human placenta and that changes in oxygen and glucose levels participate in regulation of autophagic changes in cytotrophoblast cells.

## Introduction

Autophagy is a highly regulated process involving invagination and degradation of cytoplasmic components such as damaged organelles, protein aggregates, and invading organisms through the lysosomal pathway [1]. During autophagy, proteins and organelles are sequestered into double-membrane vesicles called autophagosomes. Autophagosomes ultimately fuse with lysosomes to generate single-membrane autophagolysosomes and the sequestered material is degraded and metabolized by the cell for macromolecular synthesis and energy generation. These autophagic functions are important for cellular homeostasis and bioenergic management, and promote cell survival in response to environmental stresses such as starvation and hypoxia. 

In mammals, autophagy has been reported to be essential for pre-implantation development and in regulation of embryo survival [2]. Autophagy-related proteins such as beclin-1, LC3B, and DRAM have been shown to be present in the human placenta [3-5]. Beclin-1 is a part of an early complex that promotes synthesis and growth of pre-autophagosomal membranes [6]. LC3B is synthesized as proLC3B and converted to LC3B-I by autophagy-related proteases. Upon induction of autophagy, LC3B-I is further processed into LC3B-II and integrated into membranes of autophagosomes [6]. DRAM is a lysosomal protein that regulates autophagy in a p53-dependent manner [7]. Compared to normal pregnant women, increased autophagy has been noted in placentas from women with pregnancies complicated by fetal growth restriction (FGR) or severe preeclampsia [3-5]. In association with apoptosis, autophagy has been found to be involved in the process of membrane rupture of human amnion in term gestation [8]. Furthermore, hypoxia induces apoptotic and autophagic changes in primary human cytotrophoblasts [4,9], and there are differential changes in autophagy and apoptosis of trophoblasts between constant oxygen conditions and hypoxia-reoxygenation [10]. Notably, cytotrophoblasts transfected with *BECN1*, *LC3B*, or *DRAM* siRNA showed increased apoptosis under hypoxia compared to controls, suggesting a protective role for autophagy against hypoxia-induced trophoblast apoptosis [4]. Nevertheless, autophagic changes in human placenta throughout gestation and the mechanisms underlying the regulation of autophagy in placental development remain unclear. 

Given the fact that autophagy plays an important role in early embryo development and in pregnancy complications, the specific objectives of this study are as follows: (1) to study the extent of autophagy by characterizing changes in the levels of beclin-1, LC3B, and DRAM in the human placenta throughout gestation; (2) to ascertain whether autophagy is involved in regulation of trophoblast invasion activity using a choriocarcinoma cell line, JEG-3 cells, as a model; (3) to examine the effect of reduced oxygen and glucose on autophagic changes; and (4) to investigate the effect of reoxygenation and supplementation of glucose on autophagic changes after oxygen-glucose deprivation (OGD) in primary cytotrophoblasts obtained from normal term pregnancy. 

## Materials and Methods

This study was approved by the Institutional Review Board of Chang Gung Memorial Hospital, Taiwan (No. 98-3987B). All placental samples were collected after the subjects had provided written informed consent. 

### Collection of placental tissues

Villous samples were obtained from the following sources or scenarios: elective termination of gestations ranging from 7 to 24 weeks with documented embryonic or fetal cardiac activity by ultrasound (n = 16); pregnancies complicated by cervical incompetence (n = 2); spontaneous preterm deliveries ranging from 24 to 28 weeks in the absence of signs, symptoms, and histological evidence of chorioamnionitis (n = 2); elective repeat cesarean section (CS) prior to the onset of labor (38 to 39 weeks, n = 10); and uneventful vaginal deliveries (VD) (38 to 40 weeks, n = 10). In the current study, villous samples were grouped according to gestational age and delivery mode as follows: (1) early gestation (7–12 weeks, n = 10), (2) mid-gestation (13–28 weeks, n = 10), (3) late gestation with CS (38–39 weeks, n = 10), and (4) late gestation with VD (38–40 weeks, n = 10). Villous samples from early gestation were submitted for chromosomal study to confirm normal karyotypes. All fetuses delivered were examined by obstetricians for any gross malformations.

For mid- and late gestation, villous tissues were randomly sampled from five distinct sites from the maternal side after the placenta was delivered. Each site was midway between the cord insertion and the periphery of the placenta and midway between the chorionic and basal plates. The villous samples were quickly washed in ice-cold phosphate-buffered saline to clear the maternal blood, frozen in liquid nitrogen, and stored at -70°C for further processing. All villous samples were collected and processed within 10 min after delivery.

### Isolation and culture of cytotrophoblast cells from term placentas

Isolation and culture of cytotrophoblast cells from normal-term placentas were performed as previously described [11]. The purified cells were then plated at a minimum of 2 × 10^5^ cells/cm^2^ in 6-well plates and cultured in a humidified atmosphere with 5% CO_2_ and balanced air (standard conditions, designated as 18% O_2_ with glucose in this study). After an overnight rest, the cells were rinsed twice with pre-warmed culture medium (RPMI 1640 containing D-glucose at 2 mg/mL, 5% fetal bovine serum, antibiotics, and antimycotics; all from Invitrogen, Life Technologies, Grand Island, NY, USA) to remove non-attached cells, and the medium was changed every 24 to 48 h. 

To study the effect of reduced oxygen and glucose concentrations on the changes in autophagy-related proteins, cytotrophoblasts were cultured in a medium with or without glucose in 2% or 18% O_2_ with 5% CO_2_/balanced N_2_. After 48 h of incubation, the cell lysates were collected and stored at -70°C for further processing.

To study the effect of supplementation of oxygen and glucose on the autophagic changes after OGD, cytotrophoblasts were cultured in a medium without glucose in 2% O_2_/5% CO_2_/balanced N_2_ conditions for 48 h and then subjected to the following 4 conditions: (1) 2% O_2_ without glucose; (2) 2% O_2_ with glucose; (3) 18% O_2_ without glucose; and (4) 18% O_2_ with glucose. After 48 h, the cell lysates were collected and stored at -70°C for further processing.

### Villous explant culture

To compare the autophagic flux between villous tissues of early and late gestation, explants prepared from villous tissues of early gestation (7–10 weeks, n = 5) and from placentas of term pregnancy after elective CS (38–39 weeks, n = 5) were cultured under 2% and 8% O_2_ with 5% CO_2_ and balanced N_2_, respectively. After soaking overnight, the medium was changed, and the explants were cultured in duplicate individually with or without 200 nM of bafilomycin A1 (Sigma-Aldrich, St. Louis, MO, USA) for 48 h.

### Transfection of small interfering RNAs (siRNAs) for BECN1, LC3B, and DRAM in JEG-3 cells

Specific siRNAs for human *BECN1* (sc-29797), *LC3B* (MAP LC3β, sc-43390), and *DRAM* (sc-96209), as well as control siRNAs (sc-44230 and sc-44231), were purchased from Santa Cruz Biotechnology, Dallas, TX, USA. Transfection of siRNA was performed using the Lipofectamine 2000 transfection reagent (Invitrogen) according to the manufacturers’ instructions. siRNA transfection was performed under serum deprivation conditions, as previously detailed [4]. The siRNA/transfection reagent mixture was overlaid on the cells and incubated at 37°C in a humidified CO_2_ incubator. After 6 h, the overlaid siRNA/transfection reagent mixture was removed and replaced with fresh culture medium and incubated under standard conditions. Control siRNAs, each containing a scrambled sequence that will not lead to the specific degradation of any known cellular mRNA, were used as negative controls.

### Cell viability assay

Cell viability was determined by assessing the degree of MTT (3-[4,5-dimethylthiazol-2-yl]-2,5-diphenyltetrazoliumbromides; Sigma) reduction. At a certain time in an individual experiment, cells were incubated at 37°C in 1 mg/mL MTT solution for 2 h. After removal of the MTT solution, dimethyl sulfoxide was added, and the absorbance at a wavelength of 490 nm was recorded with a microplate reader. Cell viability was expressed as a percentage of the control culture. All experiments were performed in triplicate.

### Transwell insert invasion assay

Transwell insert invasion assays were conducted in 24-well fitted inserts with membranes (8 µm pore size; BioCoat Matrigel Invasion Chamber, BD Biosciences, Bedford, MA, USA). Briefly, JEG-3 cells were trypsinized after transfection, seeded into transwell inserts pre-coated with Matrigel at 2 × 10^5^ cells/0.5 mL per insert, and cultured under standard condition (5% CO_2_ with balanced air). Forty-eight hours later, cells were fixed and stained with Giemsa stain. Non-invaded cells on the upper surface of the membrane were removed using a cotton swab. The number of stained cells at the lower surface of the membrane was counted in at least 10 randomly selected non-overlapping fields by light microscopy. JEG-3 cells cultured at 2% O_2_ with 5% CO_2_/balanced N_2_ but without siRNA transfection throughout the experiments were used as the positive controls. All experiments were performed in triplicate and the invasion activity was expressed as the percentage of invaded cell number compared to the corresponding control.

### Real-time quantitative PCR

Real-time quantitative PCR analysis was performed as previously described [10]. Assay-on-Demand TaqMan primers and probes for *human BECN1* (Hs00186838_m1), *LC3B* (Hs00797944_s1), and *DRAM1* (Hs00218048_m1) were obtained from Applied Biosystems (Life Technologies, Grand Island, NY, USA); 18S ribosomal RNA (Hs99999901_s1) was used as an endogenous control. Thermal cycling was initiated via 2 min of incubation at 50°C, followed by an initial denaturation step of 10 min at 95°C, and then 40 cycles of 95°C for 15 s and 60°C for 1 min. All samples were analyzed on the same run, and each sample was run in triplicate. Relative quantities of *BECN1*, *LC3B*, *DRAM*, and 18S ribosomal RNA were calculated using the comparative threshold cycle method.

### Immunofluorescence

Immunofluorescence was used to determine the cellular localization of beclin-1, LC3B, DRAM, and cytokeratin 7 in the placenta at early and late gestation, as previously described [4]. Briefly, after blocking of any non-specific binding, 5-µm-thick cryosections were incubated with the following primary antibodies: rabbit anti-human beclin-1 (1:50; catalogue no. PD017, Medical and Biological Laboratories, Nagoya, Japan), rabbit anti-human LC3B (1:50; catalogue no. 2775S, Cell Signaling, Danvers, MA, USA), rabbit anti-human DRAM (1:100; catalogue no. ab68987, Abcam, Cambridge, UK) polyclonal antibodies, and mouse anti-human cytokeratin 7 monoclonal antibody (clone OV-TL12/30, 1:500; DakoCytomation, Glostrup, Denmark) at 4°C overnight. After the sections were washed, they were incubated with a cocktail of Alexa Fluor 488-conjugated goat anti-rabbit IgG and Alexa Fluor 594-conjugated goat anti-mouse IgG (10 µg/mL; Molecular Probes; Life Technologies, Grand Island, NY, USA) at room temperature for 1 h, mounted with Vectashield-DAPI (Vector Laboratories, Burlingame, CA, USA), and observed using a Leica TCS-SP2 confocal microscope (Leica Microsystems, Manheim, Germany). The negative controls used nonimmune rabbit IgG or mouse isotypic IgG instead of the primary antibody.

### Western blot

Western blotting was performed as previously detailed [4]. After individual experiments, 50 to 100 µg of cytosolic protein was separated by 12% or 16% SDS-PAGE, transferred to nitrocellulose membranes, and probed with primary antibodies against human beclin-1 (1:500, catalogue no. 3738S, Cell Signaling), LC3B (1:500, Cell Signaling), and DRAM (2 µg/mL, catalogue no. SC-98654, Santa Cruz) at 4°C overnight. The relative intensities of protein signals were normalized to the corresponding β-actin (clone AC-15, 1:10000 dilution; Sigma) density and quantified by densitometric analysis using Image J software (National Institutes of Health, Bethesda, MD; http://rsb.info.nih.gov/ij/).

### Transmission electron microscopy

Autophagy of villous tissues was confirmed by transmission electron microscopy as previously described [4].

### Statistical Analysis

Data are presented as the mean ± S.D. or median and interquartile range when data were not normally distributed. Data were analyzed and plotted using Prism 5 for Mac OS X, version 5.0d (GraphPad Software, Inc., La Jolla, CA, USA). Statistical analysis was computed using 1-way analysis of variance (ANOVA) followed by Bonferroni’s test or the Kruskal-Wallis test followed by Dunn’s multiple comparison test. In experiments studying the effect of oxygen and glucose on autophagic changes in primary cytotrophoblast cells, comparisons between groups were undertaken by 2-way ANOVA with oxygen and glucose as the main factors and oxygen × glucose as the interaction term. *P*-values < 0.05 were considered to be statistically significant.

## Results

### Levels of the autophagy-related proteins beclin-1, DRAM, and LC3B in villous tissues throughout gestation

**Figure 1:**
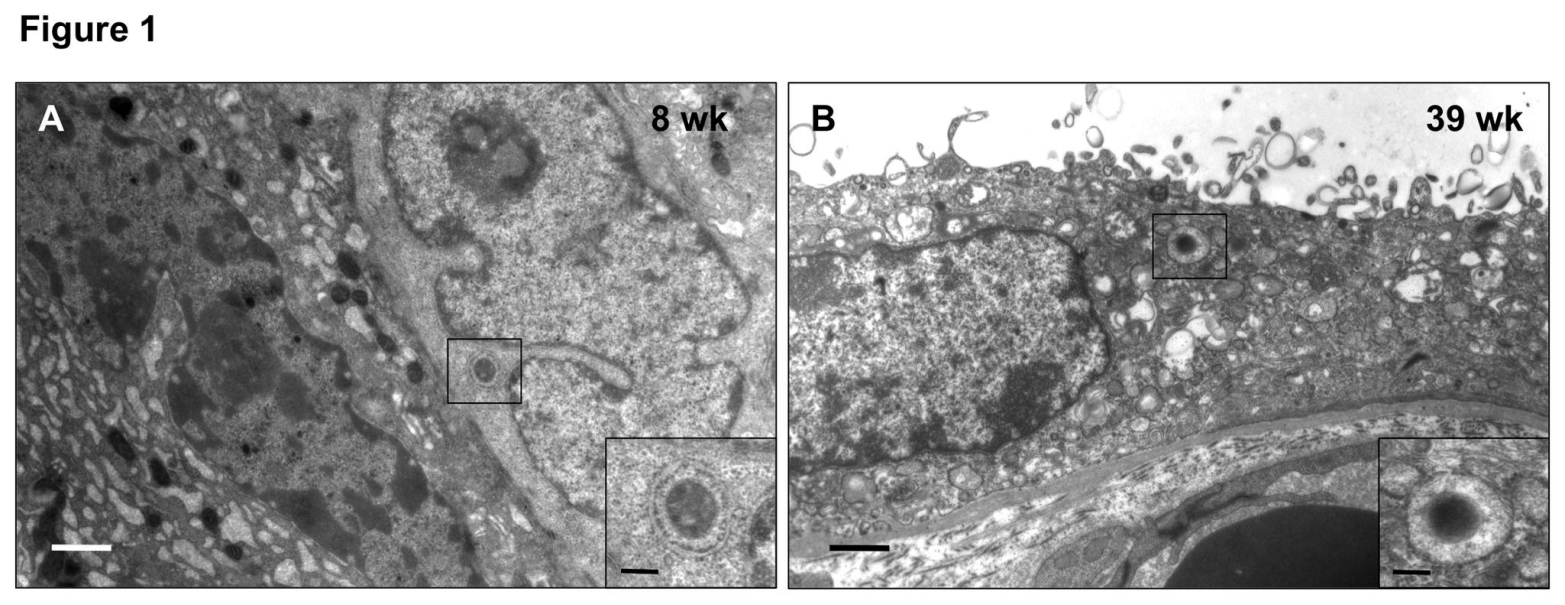
Ultrastructural assessment of autophagic vacuoles in the trophoblast layer of placentas from early and late gestation. Electron micrographs illustrating autophagic vacuoles in the villous cytotrophoblast at 8 weeks of gestation (**A**) and in the syncytiotrophoblast at 39 weeks of gestation (**B**). Scale bar = 1 µm and 250 nm in insets.

Using transmission electron microscopy, autophagic vacuoles were clearly identified in the trophoblast layer of villous tissues from early and late gestation (Figure 1). 

**Figure 2:**
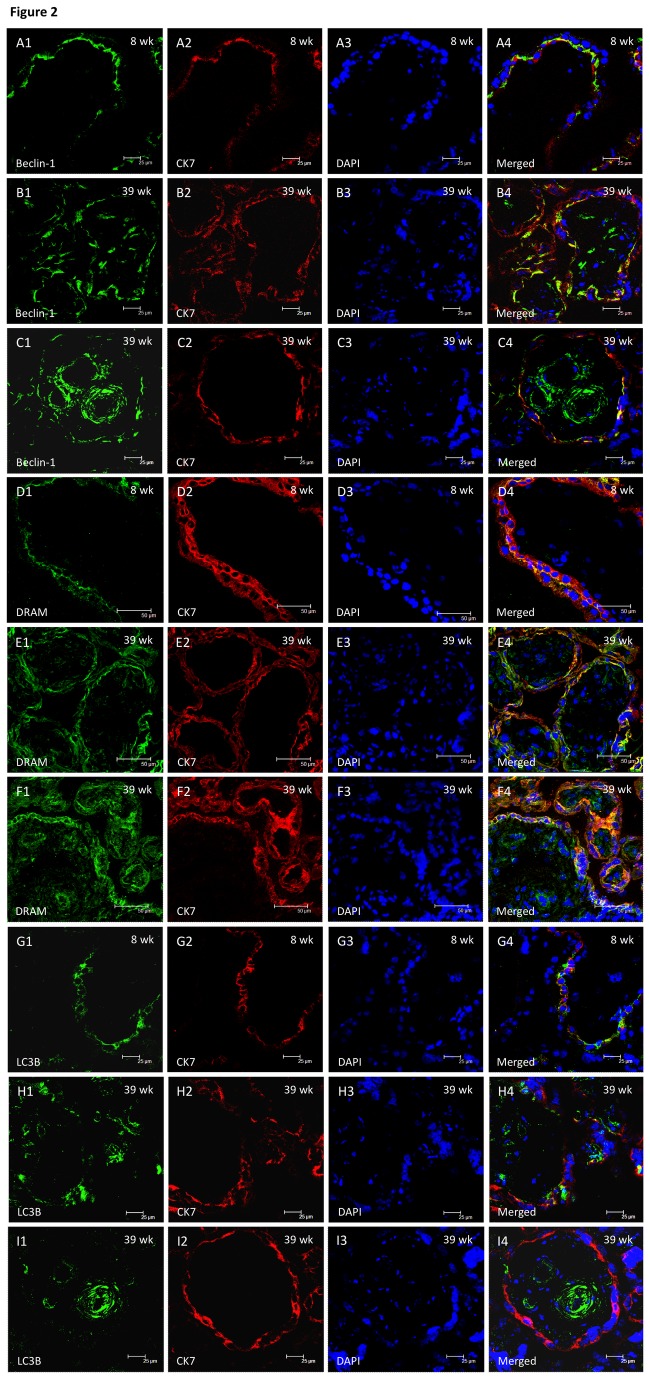
Cellular localization of beclin-1, DRAM, and LC3B in villous tissues from early and late gestation by immunofluorescence. Formation of beclin-1 and LC3B punctae was observed in the trophoblast layer of villous tissues of early and late gestation (**A1-C4 and G1-H4**), but also in the villous endothelium and adjacent smooth muscle cells, and some stromal cells in villous tissues of late gestation (**C1-C4 and I1-I4**). Immunoreactivity of DRAM was mainly localized in trophoblast cells at early and late gestation (**D1-E4**). In villous tissues of late gestation, immunofluorescence of DRAM was also noted in some stromal cells and villous endothelium (**F1-F4**). The sections were stained with cytokeratin 7 and DAPI to highlight trophoblast cells and nuclei, respectively. Scale bar = 25 μm (**A1-C4, G1-I4**) and 50 μm (**D1-F4**).

We next studied changes in 3 autophagy-related proteins, namely beclin-1, DRAM, and LC3B, to evaluate autophagic changes in placentas throughout gestation. Cellular localization of beclin-1, DRAM, and LC3B was demonstrated by immunofluorescence (Figure 2). Formation of beclin-1 and LC3B punctae was observed in the trophoblast layer of villous tissues of early and late gestation (Figures 2A1-2C4 and 2G1-H4), but also in the villous endothelium and adjacent smooth muscle cells, and some stromal cells in villous tissues of late gestation (Figures 2C1-C4 and 2I1-2I4). Immunoreactivity of DRAM was mainly localized in trophoblast cells at early and late gestation (Figures 2D1-2E4). In villous tissues of late gestation, immunofluorescence of DRAM was also noted in some stromal cells and villous endothelium (Figures 2F1-F4). 

**Figure 3:**
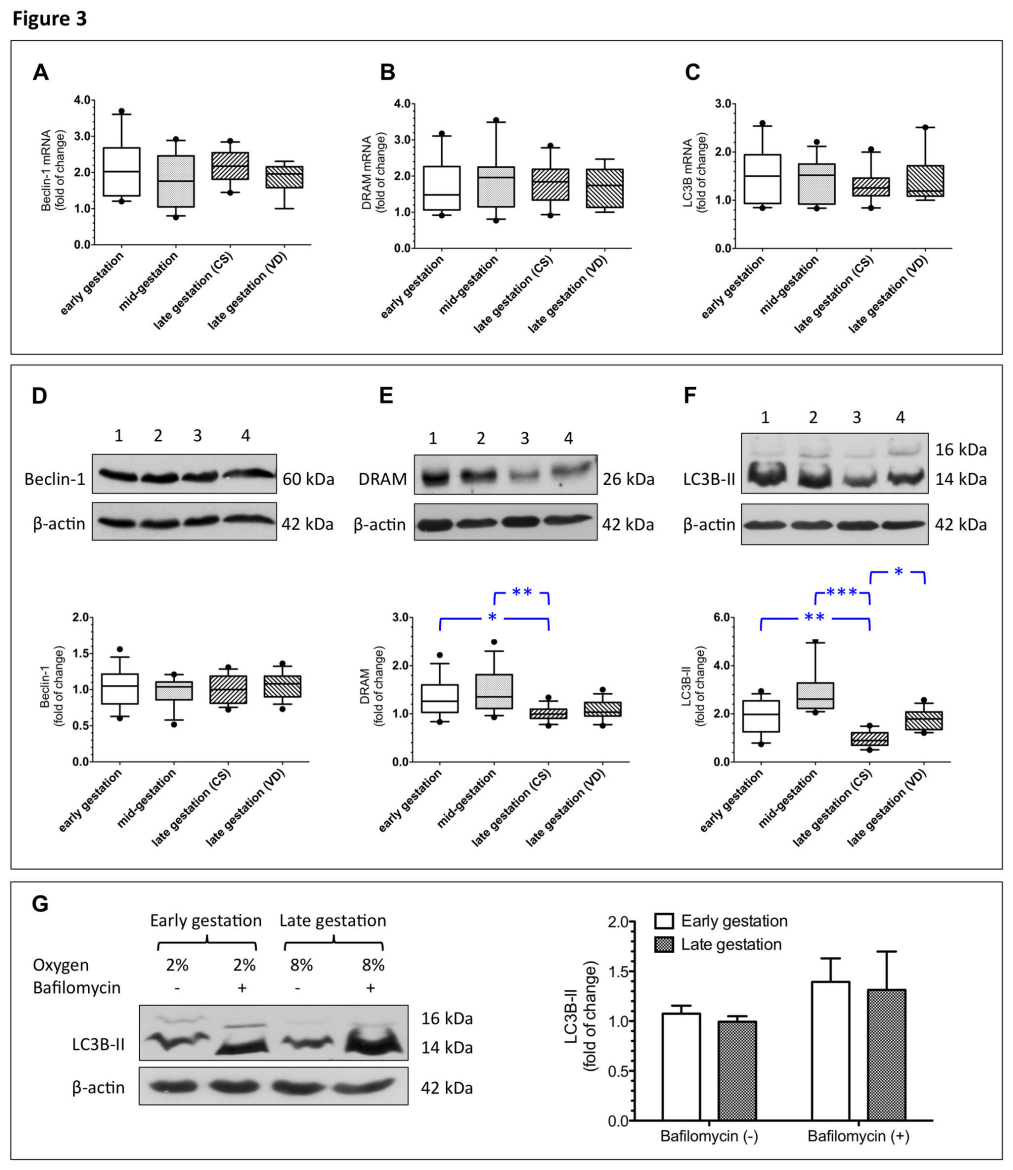
Beclin-1, DRAM, and LC3B levels and autophagic flux in villous tissues from various stages of gestation and different modes of delivery. In a total of 40 samples representing different gestational stages and modes of delivery, *BECN1*, *DRAM*, and *LC3B* mRNA were continuously expressed in villous tissues throughout gestation and there were no significant differences in the levels of *BECN1*, *DRAM*, and *LC3B* mRNA between villous tissues of different stages of gestations and between women with different modes of delivery (**A**–**C**). On the other hand, levels of beclin-1 remained constant throughout gestation (**D**), while significantly higher levels of DRAM and LC3B-II were noted in villous tissues from early and mid-gestation than in those of term placentas obtained from elective CS (**E**–**F**). Furthermore, women with VD had higher levels of LC3B-II than did those with CS (**F**). CS, cesarean section; VD, vaginal delivery. A total of 10 placentas from early gestation (7–12 weeks), 10 from mid-gestation (13–28 weeks), 10 from late gestation with CS (38–39 weeks), and 10 from late gestation with VD (38–40 weeks) were used for analysis. β-actin was used to normalize loading variability. Lane 1, villous tissues from early gestation; lane 2, villous tissues from mid-gestation; lane 3, villous tissues from late gestation with CS; and lane 4, villous tissues from late gestation with VD. Data are presented as median and interquartile range and plotted as box and whisker plots (box = interquartile range, whiskers = 90th and 10th percentiles). *P < 0.05, **P < 0.01, and ***P < 0.001, compared to villous tissues of late gestation with CS (**A**-**F**). Villous explants prepared from early gestation and from term pregnancy were cultured under 2% and 8% oxygen concentrations, respectively, with or without bafilomycin A1. Villous explants from early and late gestation showed similar levels of LC3B-II when exposed to bafilomycin A1, indicating that these two groups of tissues had comparable rates of autophagic flux under physiological oxygen concentrations. Data are presented as mean ± S.D. from 5 individual experiments (**G**).

By analyzing a total of 40 samples representing different gestational stages, we found that *BECN1*, *DRAM*, and *LC3B* mRNAs were continuously expressed in the villous tissues throughout gestation and there were no significant differences in the levels of *BECN1*, *DRAM*, and *LC3B* mRNA among villous tissues of different stages of gestations and among women with different modes of delivery (Figures 3A–3C).

Conversely, levels of beclin-1 remained constant throughout gestation, while significantly higher levels of DRAM and LC3B-II were noted in villous tissues from early and mid-gestation than in those from late gestation with CS (Figures 3D-3F). Moreover, women with VD showed higher levels of LC3B-II in villous tissues than did those with CS (Figure 3F).

### Autophagic flux in villous tissues from early and late gestation

Decreased LC3B-II levels in villous tissues of late gestation with CS can be associated with either reduced autophagosome synthesis or increased autophagosome turnover by lysosomal degradation [6]. To uncouple these effects, villous explants prepared from early and late gestation were cultured under 2% and 8% oxygen concentrations, respectively, with or without bafilomycin A1. Bafilomycin A1 is an inhibitor of autophagosome content degradation. As shown in Figure 3G, villous tissues from early and late gestation showed similar levels of LC3B-II when exposed to bafilomycin A1, indicating that these two groups of tissues had comparable rates of autophagic flux under physiological oxygen concentrations. These results also suggest that increased degradation of LC3B-II in the autophagosome in villous tissues of late gestation than in those of early gestation. 

### Autophagy and cytotrophoblast cell viability and invasion activity

**Figure 4:**
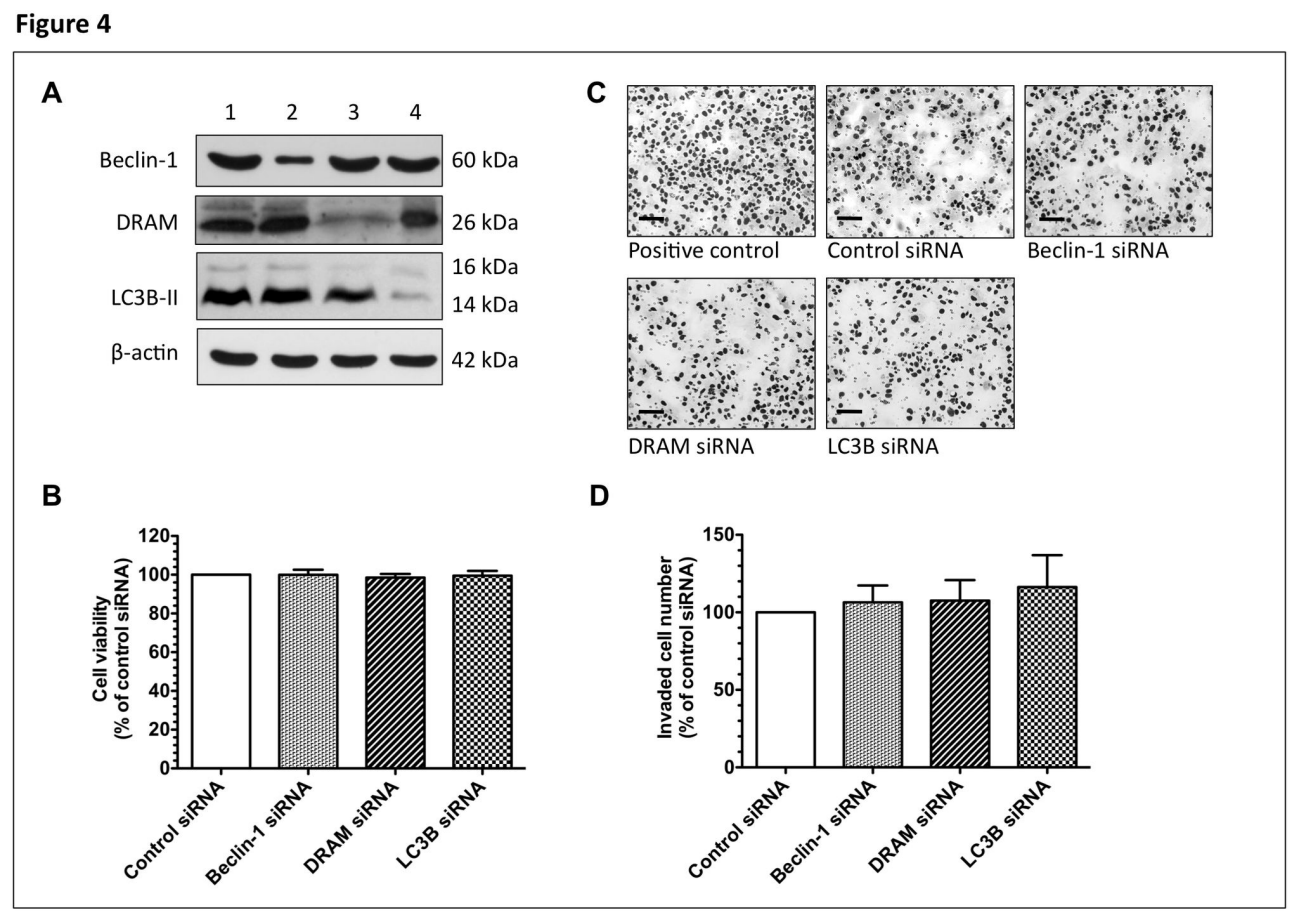
The effects of reduced autophagy on viability and invasion activity of JEG-3 cells. (**A**) Representative immunoblots for beclin-1, DRAM, and LC3B-II from JEG-3 cells transfected with or without specific siRNAs are shown. β-actin was used to normalize loading variability. Lane 1, cells transfected with control siRNAs; lane 2, cells transfected with *BECN1* siRNA; lane 3, cells transfected with *DRAM* siRNA; and lane 4, cells transfected with *LC3B* siRNA. There were no differences in viability (**B**) and invasion activity (**D**) under standard culture condition between cells transfected with control siRNAs and cells transfected with specific siRNAs. JEG-3 cells cultured at 2% O_2_ with 5% CO_2_/balanced N_2_ but without siRNA transfection throughout the experiments were used as the positive controls for the invasion assay. Scale bar = 100 μm (C). Data are presented as mean ± S.D. A total of 5 individual experiments were performed (**D**).

To further investigate the influence of autophagy on trophoblast cell behavior, we examined cell proliferation and invasion in JEG-3 cells transfected with specific siRNAs against *BECN1*, *DRAM*, and *LC3B* (Figure 4). As shown in Figure 4B, there was no difference in cell viability between cells transfected with specific siRNAs and those transfected with control siRNAs. For the invasiveness, increased invasion of JEG-3 cells was noted under 2% oxygen concentration compared to the standard condition (Figure 4C); however, there was no difference in the invasion activity under standard condition between cells transfected with siRNA against *BECN1*, *DRAM*, or *LC3B*, and cells transfected with control siRNA (Figures 4C and 4D). 

### The effects of oxygen-glucose deprivation on levels of beclin-1, DRAM, and LC3B mRNA and protein in primary cytotrophoblast cells

**Figure 5:**
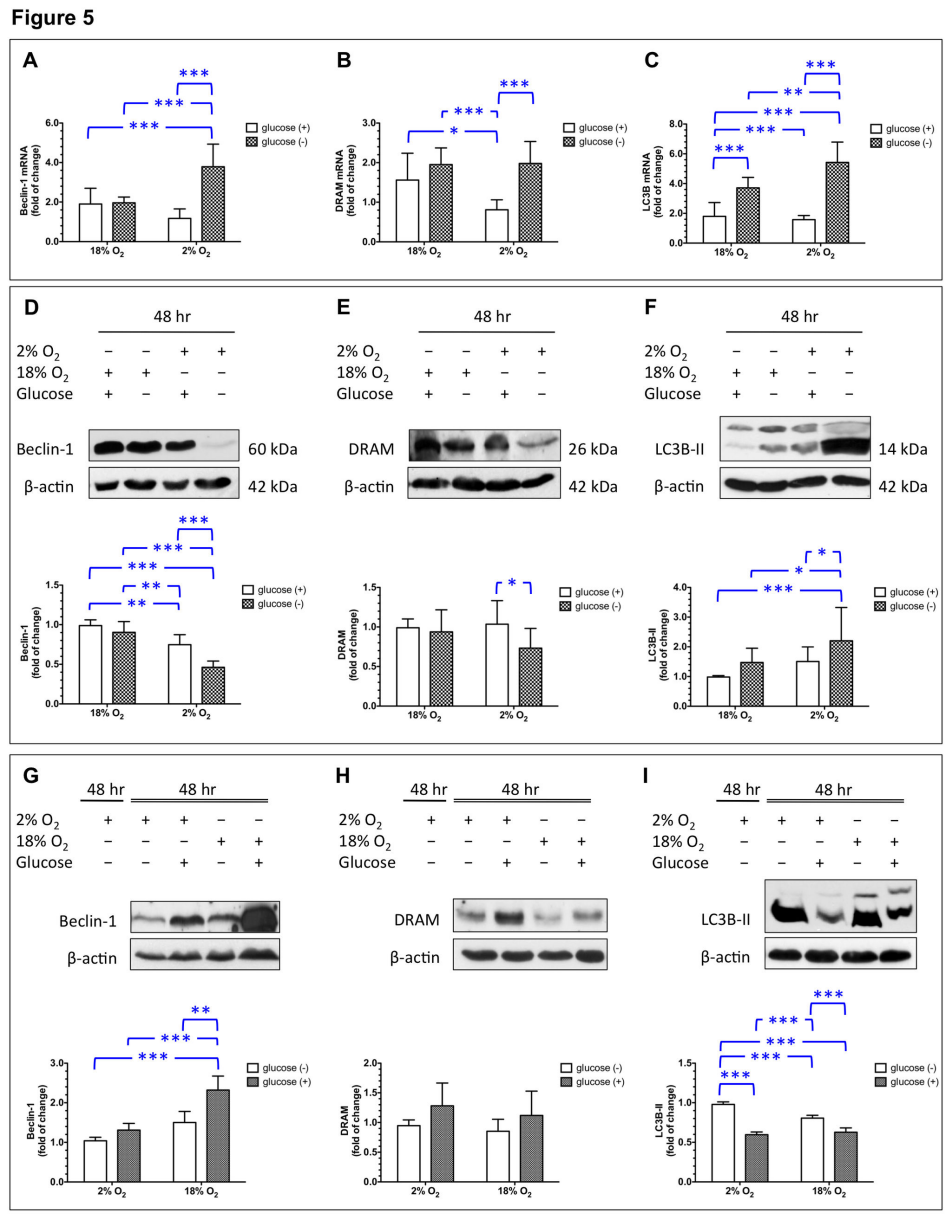
Effects of deprivation and re-supplementation of oxygen and glucose on levels of beclin-1, DRAM, and LC3B in primary cytotrophoblast cells. Cytotrophoblasts were cultured in a medium with or without glucose in 2% or 18% oxygen with 5% CO_2_/balanced N_2_ for 48 h. Data are presented as mean ± S.D. A total of 10 individual experiments were performed. *, *P* < 0.05; **, *P* < 0.01; and ***, *P* < 0.001 (**A**–**F**). Cytotrophoblasts were cultured in a medium without glucose in 2% O_2_/5% CO_2_/balanced N_2_ for 48 h and then subjected to 2% or 18% oxygen with or without glucose for another 48 h. Data are presented as mean ± S.D. A total of 10 individual experiments were performed. **, *P* < 0.01 and ***, *P* < 0.001 (**G**–**I**).

In pregnancy complications such as preeclampsia and FGR, perfusion of the intervillous space is reduced and becomes more variable due to defective transformation of the maternal spiral arteries [12,13]. We therefore studied the effects of reduced oxygen and glucose levels on autophagy in primary cytotrophoblast cells. As shown in Figures 5A and 5B, both oxygen and glucose deprivation had a statistically significant effect on mRNA levels of *BECN1* (ANOVA oxygen effect, *P* < 0.05; ANOVA glucose effect, *P* < 0.001) and *DRAM* (ANOVA oxygen effect, *P* < 0.05; ANOVA glucose effect, *P* < 0.001); however, changes in *BECN1* and *DRAM* transcription in cells cultured with supplemented glucose differed from those in cells under glucose deprivation conditions, depending on the oxygen concentration [ANOVA oxygen × glucose interaction effect, *F*(1,36) = 28.7, *P* < 0.0001 for *BECN1* and ANOVA oxygen × glucose interaction effect, *F*(1,36) = 6.0, *P* < 0.05 for *DRAM*]. In 18% oxygen, cells under glucose deprivation conditions showed similar levels of *BECN1* and *DRAM* mRNA as cells supplemented with glucose. In contrast, glucose deprivation led to significantly higher levels of beclin-1 and *DRAM* mRNA compared to glucose supplementation in 2% oxygen (*P* < 0.001).

Similarly, both oxygen and glucose deprivation had a significant effect on the levels of *LC3B* mRNA (Figure 5C, ANOVA oxygen effect, *P* < 0.05; ANOVA glucose effect, *P* < 0.001), but changes in *LC3B* transcription in cells cultured with glucose supplementation differed from those in cells with glucose deprivation, depending on the oxygen concentration [ANOVA oxygen × glucose interaction effect, *F*(1,36) = 11.2, *P* < 0.01]. Specifically, cytotrophoblast cells treated with glucose deprivation had significantly higher levels of *LC3B* mRNA than did cells cultured with glucose supplementation, with either 18% or 2% oxygen (*P* < 0.001). Furthermore, the effect of glucose deprivation on the induction of *LC3B* transcription was more profound under 2% oxygen than under 18% oxygen (*P* < 0.01). 

Figures 5D–5F show the effects of OGD on the changes of these three autophagy-related proteins, which were not consistent with the changes in mRNA levels. Statistical analysis demonstrated that both oxygen and glucose deprivation had a significant effect on the levels of beclin-1 (Figure 5D, ANOVA oxygen effect, *P* < 0.001; ANOVA glucose effect, *P* < 0.001), but these changes in cells cultured with glucose supplementation differed from those in cells cultured with glucose deprivation, depending on the oxygen concentration [ANOVA oxygen × glucose interaction effect, *F*(1,20) = 5.0, *P* < 0.05]. With 18% oxygen, cells treated with glucose deprivation had similar levels of beclin-1 as cells supplemented with glucose. In contrast, glucose deprivation led to significantly lower levels of beclin-1 than did glucose supplementation under 2% oxygen (*P* < 0.001).

For DRAM, statistical analysis demonstrated that only glucose deprivation had a significant effect on the levels of DRAM (Figure 5E, ANOVA glucose effect, *P* < 0.05), but did not show an oxygen × glucose interaction effect [*F*(1,20) = 0.08, *P* = 0.79]. In 18% oxygen, cells treated with glucose deprivation had similar levels of DRAM as cells supplemented with glucose. However, glucose deprivation led to significantly lower levels of DRAM than did glucose supplementation in 2% oxygen (*P* < 0.05).

Oxygen and glucose deprivation resulted in a different pattern of change for LC3B-II compared to the patterns of changes in the levels of beclin-1 and DRAM (Figure 5F). Specifically, both oxygen and glucose deprivation led to an increase in LC3B-II level (ANOVA oxygen effect, *P* < 0.01; ANOVA glucose effect, *P* < 0.001); however, changes in LC3B-II levels were different for cells cultured with glucose supplementation and those cultured with glucose deprivation, depending on the oxygen concentration [ANOVA oxygen × glucose interaction effect, *F*(1,20) = 25.5, *P* < 0.001]. Cytotrophoblast cells treated with glucose deprivation showed significantly higher levels of LC3B-II than did cells cultured with glucose supplementation, though a more profound effect was noted with 2% oxygen than with 18% oxygen (*P* < 0.05). 

### The effects of oxygen and glucose supplementation on levels of beclin-1, DRAM, and LC3B-II in primary cytotrophoblast cells after OGD

We next set to study the effects of reoxygenation and re-supplementation of glucose on levels of autophagic changes in primary cytotrophoblast cells after 48 h of OGD. As shown in Figure 5G, supplementation of oxygen and glucose led to a statistically significant increase in the levels of beclin-1 in cytotrophoblast cells after OGD treatment (ANOVA oxygen effect, *P* < 0.001; ANOVA glucose effect, *P* < 0.001), and a significant interaction effect between oxygen and glucose supplementation [ANOVA oxygen × glucose interaction effect, *F*(1,20) = 5.0, *P* < 0.05]. Supplementation of oxygen and glucose caused significantly increased beclin-1 levels compared to the levels in OGD controls (*P* < 0.001), and compared to the levels in cells supplemented with glucose (*P* < 0.001) or oxygen (*P* < 0.01) alone. For DRAM, supplementation of glucose after OGD, either with or without reoxygenation, seemed to increase the levels of DRAM (Figure 5H, ANOVA glucose effect, *P* = 0.03). However, there were no significant differences in the levels of DRAM between different groups, and considerable variations were noted between individual experiments. 

In contrast, the effect of oxygen and glucose supplementation after OGD on LC3B-II levels was different from that on levels of beclin-1 and DRAM (Figure 5I). Statistical analysis revealed that supplementation of oxygen and glucose led to a significant decrease in LC3B-II levels in cytotrophoblast cells after OGD treatment (ANOVA oxygen effect, *P* < 0.01; ANOVA glucose effect, *P* < 0.001), but resulted in a significant interaction effect between oxygen and glucose supplementation [ANOVA oxygen × glucose interaction effect, *F*(1,20) = 25.5, *P* < 0.001]. Compared to OGD controls, cytotrophoblast cells supplemented with glucose or oxygen only, or both, showed significantly lower levels of LC3B-II (all *P* < 0.001). In addition, supplementation of glucose had a more profound effect on the reduction of LC3B-II levels than did reoxygenation.

## Discussion

In this study, autophagy was clearly demonstrated by transmission electron microscopy and immunofluorescence in the placentas at early and late gestation. Furthermore, *BECN1*, *DRAM*, and *LC3B* were consistently expressed in the placentas, though no significant differences in protein levels of these molecules were noted in placentas between early and mid-gestation, and late gestation with VD. Together, these results indicate that autophagy is important in the development of the human placenta. 

The biological significance of autophagy in the human placenta remains unclear. One possible role of autophagy is to protect trophoblasts from apoptosis induced by stresses such as hypoxia or nutritional deprivation. The human placenta is hemochorial; maternal blood comes in direct contact with the chorion [14]. Therefore, the villous trophoblasts are easily exposed to stresses created by variable maternal blood flow. In pregnancy complications such as preeclampsia and FGR, perfusion of the intervillous space is reduced and becomes more variable due to defective transformation of the maternal spiral arteries by endovascular invasion of the extravillous cytotrophoblasts. Such profound changes in oxygen and nutritional supply may cause dysfunction of mitochondria and endoplasmic reticulum and lead to apoptosis of trophoblasts [12,13]. To avoid widespread dysregulated apoptosis, we hypothesize that the autophagic pathway is activated to maintain cellular homeostasis, thus allowing trophoblast cells to survive oxygen and glucose deprivation caused by intermittent perfusion of the intervillous space. In support of this hypothesis, increased autophagy in cytotrophoblasts, as depicted by a significant increase in the levels of LC3B-II, was noted after OGD treatment compared to the observation in cytotrophoblasts kept in standard culture conditions. Moreover, increased autophagy was found in placentas from women with severe preeclampsia and FGR compared with normal pregnant women [4,5]. 

Autophagy has also been implicated to be involved in trophoblast invasion [15], though the results are conflicting. Nakashima and co-workers found that autophagy induced by hypoxia enhanced the invasion capacity of extravillous trophoblast (EVT) cell lines [16]. In contrast, Choi and colleagues knocked down hypoxia-inducible factor (HIF)-1α with specific siRNA and showed that decreased HIF-1α was associated with increased autophagy but reduced invasion activity of HTR-8/SV-neo, an EVT cell line, under both normoxia and hypoxia [17]. Here, we found that there were no differences in viability and invasion activity under standard culture condition between JEG-3 cells transfected with specific siRNA against *BECN1*, *DRAM*, or *LC3B* and cells transfected with control siRNA. Further studies are therefore needed to clarify the role of autophagy in trophoblast invasion. 

The molecular events underlying regulation of autophagy in human placenta have yet to be fully elucidated. The HIF-1α-associated pathway has been found to be involved in the activation of autophagy induced by hypoxia [17,18]. Our observation of a more profound impact of OGD on autophagic changes than hypoxia alone suggests the presence of mechanisms other than the HIF-1-mediated pathway in the regulation of autophagy in trophoblasts. The mammalian target of rapamycin (mTOR) pathway is also important in the regulation of autophagy [18]. The mTOR pathway integrates nutritional status and stress signals, and regulation of amino acid transportation by glucose via the mTOR signaling has been observed in cultured primary cytotrophoblast cells [19]. Recently, Chen and colleagues reported that reduced mTOR activity was associated with an increased autophagic flux in primary cytotrophoblast cells [9]. Indeed, both amino acid transporter and mTOR activity are reduced in pregnancies complicated by FGR [20,21], a condition associated with increased autophagy [3,4]. 

In this study, we found that OGD caused a significant increase in the levels of *BECN1*, *DRAM*, and *LC3B* mRNA; however, the levels of beclin-1 and DRAM were lower than the levels in standard culture conditions. The discrepancy between the changes of mRNA and protein may be caused by inhibition of protein synthesis during OGD. Protein synthesis consumes a great deal of ATP. Adaptation to lowering oxygen and glucose levels requires coordinated downregulation of metabolic demand and supply to prevent a mismatch in ATP utilization and production that might culminate in a bioenergetic collapse. OGD diminishes ATP utilization by inhibiting mRNA translation and the activity of the Na-K-ATPase, and with inhibition of multiple mechanisms including mTOR signaling pathway [22]. Therefore, levels of beclin-1 and DRAM decreased during OGD, but increased when oxygen and glucose were restored. Our results also show differential changes in beclin-1, DRAM, and LC3B levels in response to changes in oxygen and glucose concentrations, indicating the complexity of interactions between these autophagy-related proteins.

In summary, our results indicate that autophagy is involved in the development of the human placenta, and that alterations in oxygen and glucose levels may participate in the regulation of autophagic changes in cytotrophoblast cells. We speculate that autophagy is important in the protection of trophoblast cells from injury caused by deficits in oxygen and glucose during pregnancy.
